# Examining public health practitioners’ perceptions and use of behavioural sciences to design health promotion interventions

**DOI:** 10.1186/s12913-023-09455-y

**Published:** 2023-05-16

**Authors:** Ariane Bélanger-Gravel, Isidora Janezic, Sophie Desroches, Marie-Claude Paquette, Frédéric Therrien, Tracie Barnett, Kim L. Lavoie, Lise Gauvin

**Affiliations:** 1 Departement of Information and Communication, Université Laval, 1055, Ave du Séminaire, Québec, G1V 0A6 Canada; 2 NUTRISS-INAF research Center, Université Laval, Quebec City, Québec Canada; 3 Research Center of the Quebec Heart and Lung Institute, Quebec City, Québec Canada; 4 School of Nutrition, Université Laval, Quebec City, Québec Canada; 5 Institut national de santé publique du Québec, Montréal, Québec Canada; 6 m361, Trois-Rivières, Québec, Canada; 7 Departement of Family Medicine, McGill University, Montréal, Québec Canada; 8 Research Center of Sainte-Justine, Montréal, Québec Canada; 9 Departement of Psychology, University of Quebec at Montreal, Montréal, Québec Canada; 10 Montreal Behavioural Medicine Center, Montréal, Québec Canada; 11 School of Public Health, University of Montreal, Montréal, Québec Canada; 12 Research Center of the Centre hospitalier universitaire de l’Université de Montréal, Montréal, Québec Canada

**Keywords:** Behavioural sciences, Public health, Knowledge translation, Qualitative study

## Abstract

**Background:**

Behavioural sciences have been shown to support the development of more effective interventions aimed at promoting healthy lifestyles. However, the operationalization of this knowledge seems to be sub-optimal in public health. Effective knowledge transfer strategies are thus needed to optimize the use of knowledge from behavioural sciences in this field. To this end, the present study examined public health practitioners’ perceptions and use of theories and frameworks from behavioural sciences to design health promotion interventions.

**Methods:**

This study adopted an exploratory qualitative design. Semi-structured interviews were conducted among 27 public health practitioners from across Canada to explore current intervention development processes, the extent to which they integrate theory and framework from behavioural sciences, and their perceptions regarding the use of this knowledge to inform intervention design. Practitioners from the public sector or non-profit/private organizations who were involved in the development of interventions aimed at promoting physical activity, healthy eating, or other healthy lifestyle habits (e.g., not smoking) were eligible to participate.

**Results:**

Public health practitioners generally agreed that behaviour change is an important goal of public health interventions. On the other hand, behavioural science theories and frameworks did not appear to be fully integrated in the design of public health interventions. The main reasons were (1) a perceived lack of fit with current professional roles and tasks; (2) a greater reliance on experiential-produced knowledge rather than academic knowledge (mainly for tailoring interventions to local setting characteristics); (3) the presence of a fragmented knowledge base; (4) the belief that theories and frameworks require too much time and resources to be operationalized; and 4) the belief that using behavioural sciences might undermine partnership building.

**Conclusions:**

This study provided valuable insights that may inform knowledge transfer strategies that could be optimally designed to support the integration of behavioural sciences theories and frameworks into public health practices.

**Supplementary Information:**

The online version contains supplementary material available at 10.1186/s12913-023-09455-y.

## Background

Since many decades, the field of health promotion significantly contributed to the public health approach to achieve better population health [1, 2]. However, the burden of unhealthy lifestyle habits (e.g., physical inactivity, poor diet, smoking, etc.) is still of concern [3–5], and theories and evidence from behavioural sciences could contribute to improving the effectiveness of health promotion interventions [6–8]. The Covid-19 pandemic has further emphasized the critical role of behaviour change to maximize population adherence to public health recommendations [9–11]. To better inform programs and policies, the World Health Organization (WHO) recently advocated for a better integration of knowledge from behavioural sciences into the field of public health [12]. Interestingly, the United Nations (UN) made a similar call to address future challenges associated with sustainable development [13]. Despite evidence of the efficacy of interventions based on behaviour change principles to promote health-related behaviours [14–17], knowledge and skills in behaviour change theories and intervention design are not always fully integrated into public health practices [8, 12, 18]. As reported by a number of behavioural scientists, it is routinely observed that behaviour change interventions are not theory-based [19–21] or that theories or behaviour change principles are not adequately operationalized [22, 23]. According to Glanz and Bishop [7], this could potentially hamper the effectiveness of interventions. Although there are still uncertainties regarding the superior effectiveness of theory-based interventions to achieve behaviour change [22], there is strong international consensus on the relevance and value of using evidence-based knowledge from behavioural sciences to inform public health programs and policies [12].

According to Brownson, Fielding and Maylahn [24], the field of public health would benefit from embracing an evidence-based approach to support better population health. However, studies on knowledge translation in this field have suggested that the uptake of research evidence and its translation into daily practice is far from being optimal in general [24–27]. This lack of uptake is not necessarily due to a lack of motivation or interest among practitioners. In a systematic review, Orton et al. [25] reported several barriers associated with the use of research evidence among public health policy makers. These included lack of fit with field realities, doubt regarding the added value of theories, negative perceptions toward research evidence, and a lack of effective knowledge translation approaches. Kneale et al. [26] also recently highlighted the predominant role of field-based evidence (e.g., the primacy of local data, the necessity to fit evidence-based knowledge with local context, and the important role of expert opinion) in comparison to research-based evidence in public health decision-making processes.

According to the knowledge-to-action framework, important steps in the design of effective knowledge translation strategies include conducting research on factors associated with knowledge uptake and use (e.g., barriers and facilitators) and ways to adapt evidence-based knowledge to practitioners’ contexts [28]. Accordingly, knowledge translation strategies need to be adapted to support better integration of evidence-based research into daily public health practices [29–31]. Although a number of studies have explored factors associated with the use of evidence-based knowledge among public health practitioners and policy makers in general [32], there is a dearth of information regarding the use of evidence-based knowledge from behavioural sciences more specifically. In a rare study on this topic, Curtis, Fulton and Brown [33] reported that public health practitioners and decision makers were sceptical regarding the added value of integrating behavioural sciences theories and evidence into public health practices. For instance, some participants believed that using behavioural sciences would lead to limited impact, mainly because of a perceived lack of fit between evidence for effectiveness from academic research and local contexts of practice. These authors also reported a professional culture that values field experience and tacit knowledge more highly than it does the applying of research evidence. They also found that practitioners’ perceived mastery of skills to apply this knowledge varied significantly according to the practitioners’ academic background. More recently, Byrne-Davis et al. [34] reported that a lack of supportive environment, time constraints, and the inherent difficulties of working within multidisciplinary teams, among others, were important barriers to integrate behavioural sciences into public health strategies to tackle the Covid-19 pandemic. Still regarding emergency responses, Weston, Ip and Amlôt [18], highlighted that difficulty in navigating through behaviour change theories was a significant barrier of their use. To our knowledge, no other study has examined factors associated with the use of behavioural sciences in public health practices. Moreover, previous studies were conducted in specific public health settings (e.g., in the UK) and for specific public health responses (e.g., managing pandemic or infectious disease outbreaks), thus limiting the possibility to generalize results to other public health contexts. Hence, the present study aims to contribute to the development of a diverse knowledge basis regarding the translation of behavioural sciences into public health. More specifically, the objective was to explore public health practitioners use of, and perceptions about theories and frameworks from behavioural sciences to design health promotion interventions.

## Methods

### Study design and sample

This study adopted a qualitative thematic analysis approach to examine public health practitioners’ current practices with regard to behaviour change and their knowledge and perceptions with respect to behavioural sciences (see the COREQ Checklist for reporting qualitative study in Supplementary Material 1)[35]. To this end, semi-structured telephone and online interviews were conducted in French (February and March 2020) and in English (fall of 2020) among a convenience sample of 27 public health practitioners working in the field of health promotion (i.e., physical activity, healthy eating, and tobacco/vaping control) in Canada. To be eligible, participants needed to be involved in the development and/or the implementation of health promotion interventions. Fourteen participants were employed in non-profit public health organizations and 13 were from the public sector. Most of the participants were female (n = 24) and French-speaking (n = 20). A snowball sampling strategy was used to recruit participants, starting from the researchers’ network of collaborators in the field of public health. These collaborators were reached out through e-mails and were invited to reach out to their collaborators and so on. This strategy was complemented by reaching out directly to a number of public health organizations involved in the promotion of physical activity and healthy eating. Because ethical considerations precluded the tracking of practitioners that were reached out through this sampling procedure, it was not possible to keep track of the number of practitioners who refused to participate and reasons why. It is worth noting that most of the interviews were conducted a few weeks before the COVID-19 outbreak in Canada. The research team was comprised of two public health practitioners and six academic researchers with a range of expertise (i.e., public health and health promotion, behavioural sciences, epidemiology, communication, and implementation sciences). The primary expertise of the principal investigator was behavioural sciences, public health, and health promotion. The study was approved by the Ethics Committee of Université Laval (approval no: 2019 − 352/03-01-2020), and informed consent was obtained from all participants. Before obtaining the participants’ consent, study background and objectives as well as data collection procedures were explained to participants. Methods were carried out in accordance with relevant guidelines and regulations of conducting research in Canada.

### Interview guide and data collection

An interview guide was developed to explore three key themes: (1) participants’ current practices regarding the design and implementation of behaviour change interventions; (2) their use and perceptions of intervention development frameworks from behavioural sciences; and (3) their use and perceptions of behaviour change theories in the design of public health interventions (see Supplementary Material 2). For the second and the third themes, they were asked to discuss their perceived challenges associated with the use of frameworks and theories from behavioural sciences. The interview guide included an opening question pertaining to their current position, day-to-day professional tasks, years of experience, and the like. The guide also included concluding questions regarding their awareness of an evidence-based and comprehensive framework to design behaviour change interventions: the Behaviour Change Wheel [36]. Awareness of this specific framework was explored for the development of future knowledge transfer activities and not for the purpose of this study; results were thus not reported. In line with our knowledge transfer focus, perceived needs for training in the behavioural sciences were explored at the end of the interviews.

Two female graduate students conducted the interviews under the supervision of the principal investigator, including one at the master’s level and the other at the doctoral level. The master’s level interviewer had a background in behavioural sciences and communication but no specific background in public health and qualitative research. The second interviewer had the same background in behavioural sciences and communication but had minimal knowledge in public health and a vast experience in conducting qualitative interviews. To ensure data quality, the master’s level interviewer was informed of the different professional tasks that public health practitioners could perform (i.e., advocating, developing and maintaining partnerships, planning and implementing interventions or supporting local organizations to do so)[37], as well as the vocabulary frequently used in this field regarding the topic of the study (e.g., using behaviour change rather than behavioural sciences). She also received brief training from the principal investigator regarding the basic principles of qualitative research (e.g., reflexivity, subjectivity, openness, etc.). Interviewers did not know participants before conducting interviews and were quite unfamiliar with the specific context of the study. Consequently, interviewers were less likely to be having oriented participants’ answers in a way that would confirm initial expectations about the results. No additional person was present during the interviews and interviews were not repeated with participants. Before data collection, a first interview was conducted to pilot test the interview guide and to ensure that the master’s level interviewer was at ease with the procedures, the vocabulary, the interview guide, etc. A number of iterations between the principal investigator and the interviewers were made throughout data collection to ensure quality. The interviews lasted between 30 and 90 min, were audio recorded and transcribed verbatim. Transcripts were double-checked by a research assistant but not returned to participants. Interviewers took notes during data collection to document any thoughts or feelings that emerged before, during, or after the interviews. The data saturation principle was applied to stop data collection. Nevertheless, data collection was carried on with all participants who volunteered to participate even though saturation seemed to emerge before the end.

### Analysis

An inductive qualitative analytic approach was adopted to classify participants’ answers in themes. According to Creswell [38], a content analysis was performed by one of the interviewers (the PhD student) in several steps. First, the coder did an initial classification to identify preliminary themes. After this first wave of coding, an iterative process was initiated with the two interviewers and the principal investigator to discuss the preliminary themes that emerged (e.g., classification, meaning, labelling, etc.). Between each meeting, the coder went back to the data and refined classification: three meetings were needed to reach consensus about the final set of themes. According to the analysis, four overreaching themes (goals of public health interventions, diversity of tasks and roles, intervention commissioning processes and the use of theories and frameworks) were identified and were then divided in sub-themes. For example, the “use of theories and frameworks” theme led to several sub-themes such as “changes in the public health paradigm”, “challenges of using theories and frameworks”, “the non-use of theories”, “the maybe-use of theories”, “experiential knowledge”, and “type of theories used”. After analysis completed, focus groups were conducted to discuss results with public health practitioners who participated in the individual interviews to validate our interpretations. All participants were invited to participate and six took part in two focus groups (French n = 3 and English n = 3). These focus groups were video recorded and transcribed verbatim. This validation step revealed that our initial interpretations were accurate, and, consequently, the previously identified themes were retained and finalized accordingly. The analysis was performed with the qualitative software *N’Vivo* (version 1.6.1). It should be noted that quotes reported in the Results section have been translated from French to English when applicable.

## Results

### Behaviour change is at the core of public health goals but…

According to the analysis, several participants agreed on the fact that, ultimately, the expected outcomes of health promotion interventions involve behaviour changes (e.g., stopping smoking, adopting a healthy diet, exercising more, etc.):

*“Well actually, sure, at the root of it, ultimately is a change in (hesitation) behaviour of the individual that we want to see*.” (P17).

Though some participants reported that they were directly involved in the implementation of behaviour change interventions, for other participants, behaviour change in the population was not the immediate expected outcome of their task:

*“Yeah, so it’s ultimately that, but in a lot of our work, we focus on interventions that change environments that will then change behaviour.”* (P10).

For instance, many participants mentioned that their role was to advocate for environmental modifications, to build partnerships, or to coordinate interdisciplinary or cross-sectoral teams. These participants considered that their role was only indirectly associated with behaviour change, or not associated at all:

*“Well yeah, we know we’re aiming for that, of course, changes in behaviour, changes in lifestyle, but we’re not going to act directly with the clientele whose behaviour we’d like to change.”* (P8).

### Knowledge, lack of knowledge, or tacit knowledge?

Knowledge of principles from behaviour change sciences seemed to be fragmented. Although some participants were initially unaware of their reliance on behavioural change theories or intervention development frameworks, they later came to the realization during the interview that they had indeed applied these principles in certain ways:

*“Well um totally yeah. Uh… I did it… instinctively, I realize as I’m talking to you that we do it, but we… we don’t make it explicit as such.”* (P9).

To illustrate this further, many of the participants who reported using theories and frameworks from the field of behavioural sciences usually referred to some elements of these theories (“intrinsic motivation,” “stages of change,” “Bandura theory”, etc.), with the caveat that this might be something that they do implicitly:

*“Uh well I’d say that Bandura is perhaps always a little bit in the back of my mind, over time we accumulate (laughs) all sorts of knowledge and it shows. I would say that it takes up mental space, a lot of space, but maybe I do so in spite of myself. It’s not necessarily always very, very conscious.”* (P3).

Overall, participants’ answers regarding their use of theories and frameworks from behavioural sciences were not always clearly articulated:

*“The main model that we use is the… theory of planned behaviour. So we’re really based on that model, mainly, I would say really for our interventions. Otherwise, at the level of… I’m working on food literacy at the moment, so we try to broaden it a little bit with the… um… social cognitive too. We’re trying to look for other models too, the Prochaska model, theories of change, but I would say that the main one is really the TPB.”* (P2).

Moreover, some participants were not able to name any theories or frameworks, although they reported that they have used them or that they were familiar with behavioural sciences:

*“Look, I know them… I can’t remember the name by heart. (Hesitation) Could you name some?”* (P11).

When asked about their usual ways of designing and implementing health promotion interventions, participants frequently reported that they follow general steps such as problem identification, field research, and partnership building, but no clear procedures informed by behavioural sciences emerged from the interviews:

*“It’s a bit like the chain we use here. You know, we explore, we define what could be done, we draw up the action plan, and then we evaluate a little. So it’s a bit of a continuum in which we [work], which is quite iterative. You can hop from one to the other, you know it’s not necessarily all the time, it doesn’t always follow this cycle, we try, but not always. So it’s like, it’s often like we plan our actions with the communities that we’re concerned about, so how can we rally the active forces? How can we move forward? Who does what? And after that, we try to move the file forward, so it’s uh, it’s like that, starting from community need.”* (P13).

Finally, though, behavioural sciences appeared to be seldom used for intervention design:

*“I’ll admit that… Ha! I think we improvise more than anything else. Well, what the hell, I … I don’t remember applying a theory of behaviour change as such.”* (P8).

### Theories need to be adapted to the “real world”

According to the participant’s views on theories and frameworks, a consensus emerged regarding their lack of fit with the implementation of field interventions:

*“I think that’s what it comes down to, because often theories look good on paper, then when it comes time to adapt a theory, wow, it’s like, I’m not sure if it’s realistic based on practice, based on reality on the ground.”* (P15)

Hence, for the majority of the participants, theories and frameworks need to be adaptable in order to be useful and implementable:

“*Again, it’s um really about figuring out… it’s people in the field figuring out how they go from a 50-page model to actually implementing it at work…. And I’m not talking about everyone, obviously, but the people that I hear from; they get overwhelmed and they panic and they don’t know what to do. And they just need someone to kind of clearly say clearly, just ask them some clear questions and help them figure out what to do next. Because if you’re not experienced… and one of the things that we know in health promotion is there tends to be a pretty high turnover and in public health in general. So you’re often working with people who have not been in their positions for very long and until recently, like Master of Public Health and stuff, that’s only been a thing for a handful of years.*” (P23)

*“Well, the challenge I would say is to follow it to the letter there. (laughs) Sometimes you can skip steps, or you can go through certain steps faster.”(P16)*.

*“But uh often we say we start with a theory and then we adjust it to our practice.”* (P15).

Also, some participants concluded that theories and frameworks are used by researchers, but not necessarily by them:

*“Uh… I find it’s mainly the researchers who use these models. Uh… my partners won’t use that stuff.”* (P16).

### Using theories is “time consuming”

Another drawback that many participants highlighted is that integrating principles from behavioural sciences into the design of their public health interventions would take too much time or would be too resource intensive. For example, participant 8 offered the following observation: “*Well, I think that just diving back into the theories… we should take the time to dive back in”*. Similarly, participant 16 highlighted tight timelines in their work, and, indirectly, suggested that using theories does not fit with their time constraints:

*“It could be because of a lack of time uh, because when one project is finished, we move on to the next one, you know.”*

Interestingly, though, one participant expressed a radically different view on this aspect, suggesting that lack of time might reflect a lack of motivation to tap into this knowledge to design an intervention:

*“Is it a question of time? So let me think, uh… so when you’re pressed for time, you’re able to get things done pretty quick. I wouldn’t put it down to the fact that we don’t have the time; I think it’s that we don’t take the time to do it because it’s something we maybe don’t like doing as much.”* (P19).

### Theories might be counter-productive for partnerships building

Finally, many participants mentioned that it is difficult to use theories and frameworks from behavioural sciences when working with partners:

*“Well, we can refer to it uh but… for sure bringing up theoretical concepts with partners is not so cool.”* (P18).

Some participants saw behaviour change theories and frameworks as potentially useful for their own conceptualization of interventions, but when working with partners and collaborators, they did not necessarily feel the need to spell these elements out:

*“Personally, in my own practice…yeah… but then, when we talk about organizations, I have difficulty imagining that I would go and present this to my board of directors and that… there’s the world of finance and the world of banks and private companies (sigh). I don’t know, maybe I’m wrong, but… these approaches [behaviour change principles] are very academic; you have to water everything down, a whole lot (laughs) when you talk to these people in any case. For my own personal practice, maybe it would help me make better choices.”* (P3).

It also emerged that some participants mainly considered the relevance of using theories and evidence from behavioural sciences with partners and collaborators that have a clinical background:

*“It’s sure that uh it also always depends on the different professionals; I mean their uh their background mm… you know what they bring to the table… I’m thinking of nutritionists who can experience the same kinds of issues in an individual way who have… you know, who can have the same vision, so yes, yes, totally.”* (P14).

Interestingly, to express the challenges associated with explicit use of a theoretical framework, one participant highlighted the fact that this would not be viewed as something positive within their own organization:

*“But you know we really don’t take the time to talk about theory and if I arrive on Monday morning and say like ‘that’s it, according to Bandura’s stages of behavioural change,’ my bosses are going to look at me, they’re going to go ‘ok there come back down to earth, come back real quick.”* (P19).

## Discussion

Overall, the findings of this study have revealed that public health practitioners interviewed generally acknowledge that behaviour change is an important goal of health promotion interventions. On the other hand, if behaviour change is seen as an endpoint goal, many public health practitioners did not perceive that behaviour change is or should be the result of their own practice, as the public health approach adopts a broader perspective on population health that does not only address changes at the individual level (e.g., environmental modification or policy building). Also, behaviour change principles do not seem to be widely known and consequently brought to bear, at least explicitly, for many reasons, including a perceived lack of fit of behaviour change theories and frameworks with professional roles and associated tasks as well as the presence of several issues associated with the use of behavioural sciences in day-to-day practices.

A perceived lack of fit between evidence-based knowledge and public health practice was already reported in previous reviews on this topic [25, 26, 39, 40]. Our results, however, do not seem to suggest that behavioural science principles were perceived as being irrelevant to public health; rather, our findings may serve to underscore the complexity of the context in which public health practitioners operate. While some participants were directly involved in behaviour change intervention design and implementation, others were mostly involved in tasks that could lead to behaviour change, but more indirectly and in the long-term. The utility of, for instance, applying this knowledge to maximize the use of new infrastructure (i.e., environmental modifications) or to support the development of an advocacy strategy was not clearly perceived by practitioners. The relevance of using behavioural sciences seems to be attributed to what could be labelled “individualistic approaches” (e.g., smoking cessation programs that include counselling). Although generalizable conclusions cannot be drawn from these results, this association of behavioural sciences with direct intervention might explain why this knowledge is not largely integrated into the field of public health. This suggests that future transfer strategies will need to integrate case studies to demonstrate how behavioural sciences could serve, for example, the development of effective public health policies. Interestingly, a recent study suggested that behavioural sciences’ theories and frameworks were largely embraced by public health practitioners [33]. However, this study included behavioural scientists that evolved in the field of public health during the Covid-19 pandemic, thus emphasizing the significant need to understand context specificities in developing knowledge transfer strategies.

The interdisciplinary nature of this field might also explain why theories and frameworks from behavioural sciences might not be regularly applied; in fact, public health practitioners, depending on their academic background and/or the culture of their organization, might rely on other types of theoretical perspectives (e.g., the socioecological model, social networks theories, participatory and community development approaches, and the like). In Canada, for instance, public health practice is predominantly guided by the socioecological approach [41], a theoretical perspective that might be dichotomously viewed as conflicting with behavioural sciences (social responsibility versus individual responsibility)[42]. Although it can be argued that principles from behavioural sciences could be used to design more effective health promotion interventions [12], knowledge transfer approaches will need to take into account the plurality of other theoretical perspectives that are guiding this field. Moreover, as the superiority of behaviour change theories over other theoretical perspectives has not been clearly demonstrated, future studies might benefit from examining the interplay between the use of theories from the field of behavioural sciences versus using theories from other fields (e.g., preferences, familiarity, etc.).

Still in line with a perceived lack of fit, some participants see behavioural sciences as more relevant for academic research and many of them mentioned that using behaviour change principles needs to be adapted to “real world” settings. This need to adapt evidence-based interventions to field settings is not new since this factor has been consistently reported as a key to overcoming lack of fit and favouring the uptake of evidence-based interventions in clinical or community-based settings [40, 43]. This is not unknown to researchers in the field of behavioural sciences as huge efforts to synthesize and simplify knowledge are currently underway. For example, a group of researchers lead by Michie and colleagues at the Centre for Behaviour Change-UCL developed the behaviour change wheel and associated tools (the behaviour change taxonomy, the COM-B model, and the theoretical domain framework, to name a few) by synthesizing a number of recognized theories and intervention development frameworks [36]. However, the uptake of these evidence-based tools in public health practice remains difficult to gauge. In the present study, participants were not aware of these innovative tools, with the exception of one participant (data not shown). In a recent study, although participants seem to be highly knowledgeable in behavioural sciences (e.g., participants reported the use of the COM-B model) they paradoxically mentioned that they would need knowledge translation tools to support the integration of behavioural sciences into practice [34]. As authors highlighted, participants did not seem to be aware of the knowledge translation tools already available. Hence, although some initiatives to favour the transfer of behaviour change knowledge in the field of public health do exist (e.g., https://www.ucl.ac.uk/behaviour-change/training; https://www.ibtnetwork.org/home/about-us/mission/), to our knowledge, the efficacy of such strategies has not been reported in the scientific literature.

Our findings also suggest that public health practitioners might have an ambivalent attitude toward the use of behavioural sciences knowledge. As mentioned above, theories and frameworks in this field were perceived as being quite rigid and more suitable for academic research. Moreover, integrating this knowledge in practice was considered to be time consuming and not particularly well suited for partnership building and consolidation. This last observation echoes what was reported by Kneale et al. [26] regarding the importance of local evidence and local actors in public health decision-making processes. The public health practitioners who participated in the present study felt that explicitly applying behaviour change theories or frameworks would unnecessarily complicate the planning process and perhaps undermine the valuable relationships that they had built with their partners over time. Similarly, Byrne-Davis et al. [34] reported significant challenges associated to the operationalization of behavioural sciences when working within teams of practitioners that come from different disciplines. This underscores the fact that public health practitioners are at the crossroads of many intervention sectors (transport, health, education, social development, housing, etc.); collaborate with stakeholders from many disciplines (e.g., ONGs, municipal or city councils, governments, researchers, etc.); and work closely with various population groups. This collaborative nature of public health practice is based upon an ability to adapt approaches and language to build and maintain relationships. Through our analysis, we have uncovered a perception that behavioural science knowledge does not help in addressing this specific challenge shared by public health practitioners. Since having a negative (or ambivalent) attitude is a significant barrier for behaviour change, perceived inconvenience associated with the use of behaviour change theories and frameworks that were reported in the present study would need to be addressed by behavioural researchers in order to promote optimal sharing of this knowledge with public health practitioners. As a result, knowledge translation tools that specifically address the challenge of building theory-based interventions in a collaborative way would be needed.

In line with previous studies, results discussed so far also highlight the importance and valued role of experiential knowledge in public health practice. As outlined by Kothari et al. [44] and further reported by Curtis et al. [33], experiential knowledge is a significant source of knowledge for public health practitioners in decision-making. As a result, knowledge translation strategies in behaviour change will need to build upon this type of knowledge to be meaningful to practitioners. However, although experiential knowledge is valuable and should be considered in capacity-building strategies, our results suggest that the prevailing knowledge base in behaviour change is somewhat fragmented. Many participants reported some form of knowledge in this field – they were thus not completely unfamiliar with behavioural sciences – but this knowledge appeared to be more implicitly used or applied in an unstructured way. This might suggest that while many (but not all) public health practitioners acquired this knowledge during their academic training – competencies in health promotion are at the core of public health training and certification in Canada)[45] – it might be hypothesized that they had less opportunity to put this knowledge into practice when entering the public health workforce. Unfortunately, relying on tacit or experiential knowledge rather than using explicit or more structured knowledge may lead to the designing of potentially ineffective (or less optimally effective) behaviour change interventions and programs [44]. In this regard, many researchers in the field of behaviour change are consistently reporting that difficulties associated with the operationalization of behaviour change theories could be associated with lack of efficacy or effectiveness [19, 22]. To address this issue, knowledge transfer strategies will need to include basic principles and models from behavioural sciences for those who want to develop a more comprehensive basis of knowledge in the field. Moreover, these strategies will need to be tailored to the background of public health practitioners, their current professional tasks in their organizations, and the context in which they work. Moreover, strategies to support the mastery of necessary skills will need to be expressed through knowledge transfer strategies to illustrate how all these notions could be used in practice [46].

This study has some limitations. First, the diversity of professional roles of public health practitioners (e.g., public sector versus non-profit organizations; organizations more closely involved in the implementation of interventions versus those more involved in environmental modifications, etc.) were not taken into account when recruiting participants. Therefore, some participants did not fully understand our questions, or their replies were somewhat off topic. On the other hand, this recruitment strategy allowed us to explore the large diversity of public health practices and to observe that behavioural sciences might, in some cases, be perceived as being perfectly aligned with practice and in other circumstances be regarded as a very poor fit. Also, relying on a purposive rather than a convenience sampling might have improved the richness of our observations. Still in line with this sampling strategy, we did not control for the organization of provenance, leading to the possibility that participants from the same organizational culture participated in the study. In this way, four participants were clustered into two different organizations. Second, interviews were conducted by two research assistants with different level of experiences in qualitative research, thus increasing the possibility that participants were prompted differently during these sessions.

The study also has several strengths. First, results were validated outside of the research team through participant-based focus groups, enabling us to validate our understanding. Moreover, interpretations stemming from our analysis were validated with the research team; a first round of validation was conducted with interviewers and the principal investigator, and a second round was carried out with the entire research team, comprising public health practitioners (MCP and FT) and behaviour change, public health, and knowledge translation researchers (ABG, SD, TB, LG and KLL). Finally, our study is among the first to report in-depth results regarding the perceptions of public health practitioners with respect to behavioural sciences. This knowledge will provide highly significant and useful information to support the sharing of such knowledge in situations of practice.

## Conclusions

To conclude, our findings suggest that while behaviour change could be ultimately what many health promotion interventions are targeting, the structured application of behavioural science tenets does not seem to be a common practice among the public health practitioners interviewed. Challenges pertaining to a perceived lack of fit and the need to adapt theories and frameworks are among the important stumbling blocks that will need to be addressed by researchers in future knowledge transfer strategies in order to share this knowledge with public health practitioners. Furthermore, the presence of a fragmented knowledge basis, the perception of complexity and difficulty in applying such knowledge as well as issues related to partnership building represent additional challenges and barriers to the use of behavioural science principles to design health promotion interventions.

## Electronic supplementary material

Below is the link to the electronic supplementary material.

Supplementary Material 1

Supplementary Material 2

## Data Availability

The datasets used and/or analysed during the current study are available from the corresponding author on reasonable request.
