# Supplementary material for: Examining public health practitioners’ perceptions and use of behavioural sciences to design health promotion interventions
Source: BMC Health Serv Res. 2023 May 16;23:493. doi: 10.1186/s12913-023-09455-y (PMC10186298; doi:10.1186/s12913-023-09455-y)
Supplement: Supplementary file 2 — Supplementary Material 2 [file 12913_2023_9455_MOESM2_ESM.docx]

**Introduction/presentation**

Hi, my name is [Name and surname]. I’m a research assistant at Université Laval and I’m working with the principal investigator of this project. Today, our meeting pertains to the research project regarding the mobilization of scientific knowledge from behavioural sciences in public health practices.

We sent you a consent form on [time of sending]. Before we start the interview, let’s take few minutes to browse it together.

--- Consent ---

**Opening questions**

1. Before we begin, tell us a little bit about what you do in the public health field. How long have you been working in this field? Which populations do you mainly work with? What topics did you work on?

**Interview questions**

2. Now, more in line with the aim of our study, tell us a bit about your experiences regarding the planning / implementation of public health interventions or prevention activities and the promotion of healthy lifestyle habits?

- What is your role and your contributions?
- Can you tell us about the different stages of planning/intervention implementation in your organization? At what moment do you intervene?
- How important would you say different methods and intervention planning guides that currently exist are in your line of work? Have you used them? If so, which ones? Tell us about your experience. Do you have any preferences? How come? What are the main challenges in working with this method?

MODEL EXAMPLES: Intervention mapping, PRECEDE-PROCEED model

3. Now, we would like to explore with you your perception of the contribution of behavioural sciences in the planning and implementation of public health actions.

- To what extent do you consider that you are ultimately aiming to change behaviour in your practice? How comfortable do you feel with mobilizing this type of knowledge?
- To what extent do you feel that the application of this knowledge serves the purpose of achieving public health objectives as part of the population responsibility approach?

a) If the participant thinks that there is a good contribution: How important do you think theories of behaviour change are in intervention planning and implementation? Have you already used them? If so, which ones? Tell us about your experience. Do you have any preferences? Why? What are the main challenges in working with this theory?

THEORY EXAMPLES: theory of planned behaviour, Bandura’s social cognitive theory, health belief model, stages of change, etc.

4. In order to plan and implement public health interventions, researchers have recently proposed the Behavior Change Wheel? Have you ever heard of this model?

1. If so, what do you know about this method and what do you think of it?
2. If the participant hasn’t heard of it: At first glance, how do you perceive this approach? How interesting does it seem?

SHORT DESCRIPTION OF THE BCW: The Behaviour Change Wheel is an approach that has been developed to support the planning and implementation of interventions in health prevention and promotion. This approach supports individual intervention planning such as the provision of health services, but also the planning of population interventions such as the implementation of regulations, legislation and fiscal measures. This approach is based on many of the existing theoretical principles and intervention development frameworks and provides a set of strategies to help operationalize (transfer to practice) theory.

5. What do you think about the possibility of having access to continuing education on the principles from behavioural sciences? Would you be interested in acquiring a training on the BCW? How relevant would this be to you?

We have now finished the interview and we would like to thank you very much for your time. Before leaving, I would like to know if you’re interested in participating in a short focus group, once the data have been analyzed. You do not have to give us an answer, but if you allow us, we would like to get your permission to contact you again. When the time comes, you can decide whether or not you want to participate.
